# Vegetarian and Vegan Diet in Fibromyalgia: A Systematic Review

**DOI:** 10.3390/ijerph18094955

**Published:** 2021-05-06

**Authors:** Yolanda Nadal-Nicolás, Laura Miralles-Amorós, María Martínez-Olcina, María Sánchez-Ortega, Juan Mora, Alejandro Martínez-Rodríguez

**Affiliations:** 1 Department of Pathology and Surgery, Faculty of Medicine, Miguel Hernández University of Elche, 03202 Elche, Spain; yolanda.nadal@umh.es; 2 Department of Analytical Chemistry, Nutrition and Food Science, Faculty of Sciences, Alicante University, 03690 Alicante, Spain; lma52@alu.ua.es (L.M.-A.); maria.martinezolcina@ua.es (M.M.-O.); mso20@alu.ua.es (M.S.-O.); juan.mora@ua.es (J.M.); 3 Alicante Institute for Health and Biomedical Research (ISABIAL), 03010 Alicante, Spain

**Keywords:** fibromyalgia, vegetarian diet, veganism, diet therapy, chronic diseases, nutrition, public health, dietary supplements

## Abstract

Fibromyalgia (FM) is a chronic non-degenerative disease characterized by the presence of multiple symptoms such as chronic pain, which negatively influence the quality of life of sufferers, most of whom are women. Currently, there is no effective treatment to limit the impact of these symptoms. The aim of this research is to review the scientific evidence on the effect of following a vegetarian or vegan diet on fibromyalgia patients. A systematic review included the original articles that answered the research question. These articles were in 2021 in the PubMed, Web of Science and Scopus databases. The research used the PRISMA (preferred reporting items for systematic reviews and meta-analyses) guidelines. No time restriction was applied, and grey literature was not included. The evaluation of the methodological quality of the articles was carried out using the following different scales: STROBE (strengthening the reporting of observational studies in epidemiology), PEDro (Physiotherapy Evidence Database), and MMAT (Mixed Methods Appraisal Tool) scales. A total of 88 studies were analyzed, of which 6 investigations were included in this systematic review (*n* = 4 clinical trials and *n* = 2 cohort studies). These investigations show significant improvements in biochemical parameters, quality of life, quality of sleep, pain at rest and general health status when following mainly plant-based dietary patterns. In conclusion, these findings are promising but interpretation of the findings is limited due to the methodological quality of the studies. Well-designed clinical trials are needed to consolidate these dietary recommendations in FM patients.

## 1. Introduction

Fibromyalgia (FM) is a chronic non-degenerative disease of unknown etiology without effective medical treatment that mostly affects women [1,2]. At present, the diagnostic and classification criteria are still under debate. As a result, the treatments for FM are also under investigation [3]. No single treatment has achieved a reduction in the symptoms [4]. The prevalence of FM has not been determined using a large or international population base, but it is commonly estimated to affect 1–3% of the population [5]. FM is the third most common musculoskeletal disease, and its occurrence increases with age [3].

The experienced pain not only reduces overall quality of life but also has a significant impact on basic functions such as sleep and cognitive ability [6]. It is often accompanied by other characteristic symptoms, such as fatigue or morning stiffness, headaches, irritable bowel syndrome, anxiety, or depression [7,8].

Dietary behavior and dietary intake are lifestyle factors that might influence the occurrence, maintenance, and perception of chronic musculoskeletal pain [9]. The lack of an effective treatment has led FM patients to question whether dietary changes can bring about improvements [4]. Therefore, it is of a crucial relevance to review what is known and to investigate what else could be done to improve health and quality of life in these patients [5]. More recent scientific evidence has found that some treatments such as pharmacotherapy, psychological therapies, patient education, physiotherapy, and dietary interventions are effective to reduce symptoms. [6,10,11,12,13,14].

A healthy diet is essential, especially in patients with chronic diseases [15]. In the omnivorous western diet, foods high in pro-inflammatory nutrients such as glutamate are increasingly found [16]. These compounds aggravate the symptoms of FM [16]. A diet rich in antioxidants such as a plant-based diet (vegetarian and vegan diet) helps to alleviate these symptoms [4,16]. The American Dietetic Association states that vegetarian or vegan diets are healthy, nutritionally adequate and provide health benefits in the prevention and treatment of diseases [17,18,19].

A vegetarian diet consists of not eating meat, fish, or poultry. However, the eating patterns of vegetarians can be very different. A lacto-ovo-vegetarian is characterized by a diet based on grains, vegetables, fruits, legumes, seeds, nuts, dairy products, and eggs, but excludes meat, fish, and poultry. The lacto-vegetarian excludes eggs, as well as meat, fish, and poultry. The vegan, or pure vegetarian diet is like the lacto-vegetarian pattern, with the added exclusion of dairy and other animal products [18,19].

Plant-based diets are characterized by lower levels of saturated fat, cholesterol, and better control of serum glucose. In addition, high intakes of plant foods and limited amounts of animal foods provide fiber, magnesium, potassium, boron, folate, and antioxidants such as vitamins C and E, carotenoids, and phytochemicals. However, vegan diets may contain lower than recommended intakes of vitamin B-12, vitamin D, calcium, zinc and sometimes riboflavin [18,19,20].

Scientific evidence supports that vegetarian diets decrease the relative risk of many chronic diseases [21]. Following a vegetarian diet helps to reduce the incidence of high blood pressure, cardiovascular disease, stroke and metabolic syndrome [22]. It also has a protective effect against the incidence and mortality of ischemic heart disease and cancer [23]. Fibromyalgia is strongly associated with inflammation [13]. A vegetarian diet could be a useful method to control inflammation [24,25].

Haghighatdoost et al. [24] found in their meta-analysis that vegetarianism is associated with lower serum C-reactive protein concentrations, when people follow a vegetarian diet for at least 2 years. Similarly, Craddock et al. [26] found that vegetarian patterns were associated with decreased plasma concentrations of C-reactive protein, fibrinogen and total leukocytes. These results are consistent with the research conducted by Aleksandrova et al. [25] which found an association between plant-based diets and lower levels of inflammation and oxidative stress [25].

Several reviews have shown that vegetarian diets lead to greater loss of weight, visceral fat, blood lipids, oxidative stress markers and medication in some chronic diseases (such as type 2 diabetes, chronic kidney disease or rheumatic diseases) compared to diets without food restrictions [15,27,28].

Gluba-Brzózka et al. [15] reviewed the effect of vegetarian diets on chronic diseases, specifically kidney disease. This review found that patients who followed vegetarian diets obtained adequate nutrition, and cardiovascular and functional benefits [15]. On the other hand, Hänninen et al. [27] concluded that subjects consuming plant foods have increased levels of carotenoids and vitamins C and E, and reduced serum cholesterol concentrations. In addition, they observed that subjects on a vegan diet had decreased body weight, joint pain and quality of life compared to omnivores [27].

Kahleova et al. [28] examined the effect of a vegan diet in type 2 diabetic patients. The results they found in the review were a decrease in weight, plasma glucose, HbA1c, lipids, visceral fat, markers of oxidative stress and hypoglycemic medication due to lower insulin resistance in those patients who followed a plant-based diet versus those who followed an omnivorous diet. All three of the above-mentioned reviews [15,27,28] concluded that plant-based diets decreased health risk factors for chronic diseases.

It has therefore been hypothesized that a mainly plant-based diet such as a vegetarian and vegan diet provides some beneficial effects for chronic diseases, improving the severity of symptoms suffered in FM. The aim of this systematic review is to evaluate the efficacy of mainly plant-based diets such as vegan and vegetarian diets (intervention) in patients with FM (population) compared to omnivorous diets (comparation), and to examine the main effects of these diets on patients’ symptoms and the improvement in their quality of life (outcomes).

## 2. Materials and Methods

### 2.1. Design

This systematic review has been carried out following the recommendations of the preferred reporting items for systematic reviews and meta-analysis (PRISMA) statement and guidelines for systematic reviews [29,30]. The registration number of this systematic review in the PROSPERO platform is CRD42021245536.

### 2.2. Eligibility Criteria

Inclusion criteria were made following the selection protocol based on the population, intervention, comparison, and outcome (PICO) questions. Any study that recruited people with FM (population) and looked at the effect of a mainly vegetarian, vegetarian, or vegan diet (outcome) versus an omnivorous or western diet (comparison), was selected for inclusion in the systematic review. The presence of FM was to be assessed according to the American College of Rheumatology (ACR) criteria or under medical diagnosis [31]. Exclusion criteria were articles in which the subjects did not suffer from FM or in which the nutritional intervention was not a mainly plant-based, vegetarian, or vegan diet. Papers were limited to journal articles and to the English or Spanish language. No studies were excluded because of the years covered or the status of the population.

### 2.3. Search Strategy

A search strategy was used to find studies linking mainly vegetarian, vegetarian, and vegan diets in FM patients. The following databases were searched on 20 January 2021: PubMed, Scopus, and Web of Science. The keywords for fibromyalgia were “fibromyalgia”, “fibrositis”, “FMS”, “fibromyalgia [MeSH Terms]”, for vegetarian diet were “Diet, vegetarian [MeSH Terms]”, “vegetarian*”, “lactovegetarian*”, “lacto-vegetarian*”, “vegetarianism*”, “lacto-ovovegetarian*”, “lactoovovegetarian*”, and for vegan diet were “vegan*”, “veganism*”, “Diet, vegan [MeSH Terms]”, “vegans [MeSH Terms]”.

In PubMed, the search strategy used was as follows: (((diet, vegetarian [MeSH Terms]) OR vegetarian* OR lactovegetarian* OR lacto-ovovegetarian* OR lactoovovegetarian* OR lacto-vegetarian* OR vegetarianism) OR (vegan* OR veganism OR (diet, vegan[MeSH Terms]) OR (vegans[MeSH Terms]))) AND (fibromyalgia OR fibrositis OR FMS OR fibromyalgia[MeSH Terms]). The search strategy was adapted to each database, the same keywords and Boolean descriptors were used in all of them. No filters were applied in the different databases. All articles obtained with the established search strategy were analyzed, duplicates were removed. An additional hand search was carried out on the bibliographic references of systematic reviews covering nutrition, including one new study not found in the databases.

### 2.4. Data Collection

The data collection was carried out following the research protocols [29,30]. A careful reading was carried out to confirm that the research question and inclusion criteria were met in the articles found. The search and critical reading were carried out by two authors independently (Y.N.-N. and L.M.-A.) and compiled with a reference management software.

### 2.5. Data Synthesis

Two independent researchers performed and compared the data extraction (Y.N.-N. and L.M.-A.). In case of any discrepancies, a third researcher (A.M.-R.) resolved them. A data sheet to record the most relevant information of the included research was prepared with the following variables: authors, year, and country in which the research was carried out, study design, impact of the journal in which it was published, study population, intervention groups, existence of a control group, research objective and hypothesis, tools used for data collection, and relevant findings.

### 2.6. Methodological Quality

A final analysis was carried out independently by two researchers (Y.N.-N. and L.M.-A.) to assess the methodological quality of the full texts that met the eligibility criteria. Depending on the type of research study, different tools were used. The papers included in this review are analytical cohort studies or clinical trials, and the scales used were as follows: STROBE checklist (strengthening the reporting of observational studies in epidemiology) [32], PEDro (Physiotherapy Evidence Database) verification scale [33], and MMAT checklist (mixed methods appraisal tool) [34]. The results of this critical appraisal can be found in the supplement to this paper (Tables S1–S3). All articles considered “low quality” were excluded from the review.

## 3. Results

A total of 88 studies were collected, 33 were excluded due to duplication. Of the 56 selected studies, 38 were excluded because they were not relevant to the study (*n* = 27), diseases other than FM (*n* = 6), book or chapter (*n* = 2), patents (*n* = 1) and conference proceedings (*n* = 2).

The full text of the remaining 18 articles was analyzed, among which 12 were excluded for the following reasons: systematic reviews (*n* = 9), the incorrect language (*n* = 1), the full article was not available (*n* = 1) and the dietary intervention was one other than a vegetarian or vegan diet (*n* = 1).

**Figure 1:**
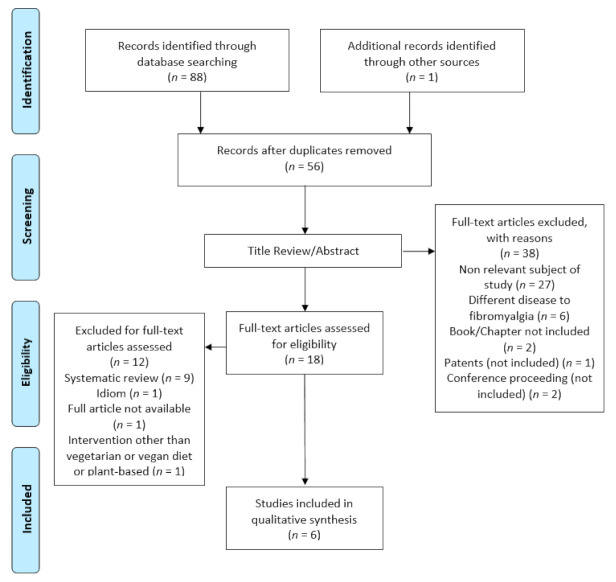
PRISMA diagram: Flowchart of study section process.

A total of six studies were included. These results can be found in the evidence search and selection summary, which is based on the PRISMA flowchart (Figure 1).

### 3.1. Overview of Included Studies

From the six studies included in this review, four were clinical trials [35,36,37,38] and two were observational cohort studies [8,39]. The selected studies included 157 FM patients in both the intervention and control groups, and more than 117 were women. The follow-up time ranged from 3 weeks to 7 months.

All the studies included different dietary interventions, as follows: a vegan diet with all food uncooked [35]; a pure vegetarian diet, mainly raw [8]; an isocaloric lacto-vegetarian diet [36]; a normocaloric mainly vegetarian modified whole grain Mediterranean diet [39]; a vegan diet [38]; and a vegetarian diet [37]. In addition, two clinical trials also included other concomitant physical therapies [36,39]. The studies used different methods to evaluate the effect of the intervention. Table 1 summarizes the characteristics of each intervention, in terms of the type of study, participants, interventions and results.

### 3.2. Effect on Biochemical Parameters and Biomarkers

Four of the included studies [35,37,38,39] examined the effect of a vegetarian and vegan diet on analytical parameters. Kaartinen et al. [35] found that a living food (LF) diet (i.e., uncooked vegan diet) improved serum total cholesterol (*p* = 0.003), but found no significant changes as a function of dietary pattern in the erythrocyte sedimentation rate (ESR) (*p* = 0.154) and hematocrit (*p* = 0.184). Hanninen et al. [38] showed that subjects following an LF diet significantly increased their levels of beta- and alpha-carotene as well as lycopene and lutein compared to the control groups. Michalsen et al. [39] analyzed stool to test whether improvements in fecal flora through a mainly vegetarian diet led to an improvement in FM symptoms. However, the nutritional interventions were not associated with the changes in stool analysis over the three months of the intervention. Neither did they find correlations between the disease course and immunoglobulin A (IgA) concentrations [39]. Hostmark et al. [37] aimed to analyze plasma fibrinogen, serum peroxides, lipids and apolipoproteins in FM patients following a vegetarian diet. A significant decrease in the serum peroxide levels was found, decreasing from 3.60 ± 0.14 to 2.82 ± 0.15 µmol/L [37]. Similarly, the total serum cholesterol and fibrinogen concentration decreased in all the participants [37]. The apolipoproteins also decreased, with a 26% decrease in apolipoprotein B and a 13% decrease in apolipoprotein A [37]. Finally, serum triglyceride values were not altered by following a vegetarian diet [37].

### 3.3. Effects on Quality of Life and Health Status

Two of the six studies included in this review evaluated the effect of raw vegan diets on quality of life and general health status. Kaartinen et al. [35] analyzed the influence of the LF diet on depression using the Beck depression inventory (BDI) questionnaire [40]. No significant differences (*p* = 0.112) were found between the different dietary patterns. They also analyzed the influence of diet on physical exercise tests but found no significant changes between the groups [35]. However, Donaldson et al. [8] did find significant improvements in physical tests (*p* < 0.03), although these tests were more related to physical movement skills. Donaldson [8] also found positive results on the SF-26 questionnaire [41]. Physical functioning, physical role, general health, vitality, social functioning, emotional role, and mental health improved in patients with the plant-based dietary intervention (*p* < 0.01). Quality of life was assessed using the QOLS questionnaire [42]. A significant improvement (*p* for trend <0.01) in this parameter was observed in those patients with an LF diet [8]. After 7 months of intervention, in four of the seven scales that make up this questionnaire, no differences were found between the FM and healthy subjects [8].

### 3.4. Effects on Body Weight and Body Mass Index

Due to the known relationship between body weight and FM symptoms [43], four investigations included these variables in their analysis. Kaartinen et al. [35] showed that following a raw vegan diet significantly decreased body weight and body mass index (BMI) (*p* = 0.0001). Furthermore, they observed that after the intervention period, where the patients returned to their usual dietary patterns, their BMI increased significantly. Martínez-Rodríguez et al. [36] observed that following a lacto-vegetarian diet and an exercise plan based on core stabilization decreases fat mass content (% and kg) and increases fat-free mass content (% and kg). This research also included an intervention group with a placebo exercise program. In this group, where the only intervention was a lacto-vegetarian diet, no significant differences in body composition were found. However, in the control group where there was neither a dietary nor physical intervention, a significant increase in the total body mass and fat mass (% and kg) was found [36]. Consistent with these results, the study conducted by Michalsen et al. [39] also found no significant differences in those patients who followed a vegetarian diet. Finally, Hostmark et al. [37] did find significant differences in body weight before and after the 3-week vegetarian diet intervention.

### 3.5. Effects on Fibromyalgia Symptoms

Among the six studies selected in this review, four of them specifically analyzed the effect of plant-based diets on FM symptoms using different questionnaires and tests. Kaartinen et al. [35] showed that following an LF diet significantly decreased pain at rest (*p* = 0.005). This positive effect disappeared as animal foods were reintroduced [35]. In addition, an improvement in sleep quality (*p* = 0.0001), reduction in morning stiffness (*p* = 0.000001) and in the rheumatologist’s overall questionnaire (*p* = 0.038) was found. Regarding the impact of FM on the person’s life, measured through the FIQ questionnaire [44] in the research of Donaldson et al., a considerable decrease (*p* < 0.05) was observed in those patients who followed a pure raw vegetarian dietary pattern [8]. As regarding to the VAS (visual analogue scale) [45], Martínez-Rodríguez et al. [36] found a decrease in somatic pain intensity when a lacto-vegetarian diet was applied together with the core stabilization program; no differences were found without the sport intervention and increasing in the control group. Michalsen et al. [39] also used this scale to evaluate the severity of pain after the intervention, a decrease in intensity was found in those patients who followed a fasting, but it was not significant in respect to those who followed a vegetarian diet.

Hanninen et al. [38] found an improvement in joint stiffness (*p* = 0.001) and pain (measured by the VAS scale) (*p* = 0.003) in those patients who followed an LF diet during the intervention.

## 4. Discussion

The present study has reviewed all current scientific evidence regarding to the use and effects of mainly plant-based diets (vegetarian and vegan diets) on the symptoms and quality of life of FM patients. Six studies were identified that met the inclusion and methodological quality criteria. According to the analyzed articles, a mainly plant-based diet improves biochemical parameters such as total cholesterol, peroxidases and fibrinogen, body weight, quality of life, pain at rest as well as other symptoms of FM and their impact on health. Given that the symptoms of FM start from different physiological points (metabolic alterations, hypothalamic axis, cortisol, oxidative stress and other changes in the central nervous system), these dietary interventions should be combined with other multidisciplinary treatments to improve the symptoms of FM and the quality of life of patients [46,47,48,49,50,51].

Vegetarian and vegan diets are based on significant amounts of plant-based foods. These provide high levels of nutrients such as fiber, vitamins, minerals and antioxidants. This information led to the main hypothesis of the study, in which they were expected to improve FM symptoms due to their anti-inflammatory properties [5,52]. The studies included in this review have shown a significant improvement on quality of life [8,35], pain [35,36,38], sleep quality [35] psychological disturbances such as anxiety and depression [35] and general health status [8,35,38].

Elevated BMI levels have been directly linked to increased pain and functional status in FM patients [43]. In addition, Senna et al. [53] and Barnard et al. [54] found that there is a positive impact between weight reduction and decreased inflammation.

Studies by Hostmark et al. [37] and Kaartinen et al. [35] showed that following a vegetarian or vegan diet for several weeks resulted in a decrease in body weight. However, Michalsen et al. [39] found no significant difference in weight following a mainly vegetarian diet and additional physical exercise therapy. Martínez-Rodríguez et al. [36] performed a randomized clinical trial in which they showed that following an isocaloric lacto-vegetarian diet accompanied by core stabilization exercises decreased body composition. However, the intervention group based on the isocaloric lacto-vegetarian diet alone had no significant differences in body composition, this was due to the fact that no training program or calorie deficit was performed [36].

Therefore, these studies show that in order to achieve an improvement in body composition through a vegetarian or vegan diet, it is necessary to follow physical exercise guidelines and an energy deficit. In these patients with FM, it is of great importance to reduce body weight and fat mass, due to the fact that adipocytes synthesize inflammatory markers such as cytokines that help to maintain body pain [55].

In terms of symptom improvements, this review has found that following an LF diet (i.e., an uncooked vegan diet) produced an improvement in sleep quality, decreased morning stiffness, chronic pain and improved general health, especially in physical functioning, physical role, general health, vitality, social functioning, emotional role, and mental health [8,35,36,38]. However, Michalsen et al. [39] could not demonstrate a significant improvement of symptom severity in those FM patients following a mainly vegetarian diet or fasting. This may be because some of the participants included animal products on a weekly basis [16,20,24].

The benefits of these dietary patterns in FM symptoms are mainly due to the nutrient supply they provide. These diets consist mainly of vegetables, fruits, nuts and seeds, mushrooms, legumes and whole grains. Therefore, they provide high levels of antioxidant vitamins (such as vitamin C and vitamin E), minerals, fiber and other components such as resveratrol and polyphenols compared to the omnivorous diet [56]. These nutrients counteract the oxidative stress produced by inadequate nutrition [57,58] and lead to an improvement in FM symptoms [4,59].

These modifications in health are observed in researches such as those by Kaartinen et al. [35] and Hanninen et al. [38] where they conclude that fibromyalgia patients who follow a vegetarian diet have an improved quality of life compared to the omnivorous diet group. Furthermore, in several articles studied [35,38] it has been seen that once the intervention with a vegetarian or vegan diet ended, the beneficial effects disappeared over time when returning to the usual omnivorous diet. These results should be taken into account when assessing this nutritional treatment as a solution to reduce the symptoms of FM, since if adherence is not adequate over time, the improvement in the quality of life may not be enhanced on a long-term perspective [35,38].

The main limitation of this study is that only six studies have examined the relationship between a plant-based diet and fibromyalgia. The limitations of the studies in this review should be kept in mind as they may limit the extrapolation of the results. Only three of the six studies finally included had a control group [35,36,38], and only one of them [36] randomized the division of the sample. None of the studies performed a double-blind intervention, as it is a dietary intervention that includes dietary restrictions, so there is less control over the presence of confounding variables that influence the results. In addition, the studies were composed of a relatively small sample number, between 10 and 35 FM participants.

The time spent on each intervention ranged from 3 weeks to 3 months. This diversity in the follow-up of the interventions influenced the ability to observe the effect of the treatments. However, several [8,35,38,39] studies did analyze the effects months after the end of the intervention, so the consistency of these results are greater. A further limitation found when analyzing the results was that each study assessed symptom improvement with different scales and tests, which means that they cannot be compared with each other.

A strength of the studies analyzed is that the degree of adherence to the dietary pattern of the intervention was assessed and was high in all of them, so the results were not biased by this factor. Adherence to the diet was verified by dietary records and urinary sodium analysis [6,8,13,35,36,37,38,39].

In addition, more studies are needed to consider confounding variables such as gender, degree of pain and influence of medication used on the results. However, even considering that most studies are at risk of bias, the quality of the studies was good and an improvement in pain and a decrease in FM symptoms was observed in those patients who followed a mainly plant-based diet such as vegetarian or vegan [6,8,13,35,36,37,38,39].

The findings of these interventions are potential and promising, although more research with well-designed studies is needed to establish these dietary interventions as the nutritional treatment of choice for patients with FM.

## 5. Conclusions

Mainly plant-based diets such as vegetarian or vegan diets seem to reduce FM symptoms and improve the quality of life of these patients. Body composition, sleep quality, depression and body inflammation have improved following these dietary patterns. However, these conclusions are not robust due to the limited quality of the studies done to date.

Future research in FM patients’ needs to be well designed in order to firmly conclude the effect of these promising dietary interventions. These dietary treatments would fit into the multidisciplinary treatment of FM with positive outcomes on patients’ lives.

## Figures and Tables

**Table 1:** Vegetarian or vegan interventions in fibromyalgia patients.

Study	Type of Study	Participants	Interventions	Results
Kaartinen K, 2000 [35]	Clinical trial	28 females with FM (average age 51–52 years); intervention group (*n* = 15), control group (*n* = 13)	- Intervention group: “LF diet” (vegan diet with all food uncooked). Supplement of vitamin B12. - Control group: Unrestricted diet	- Laboratory parameters—cholesterol, ESR, hematocrit: improvement in serum cholesterol in the LF group. No significant changes in ESR or hematocrit in either group. - Body weight: significant decrease in body weight and therefore BMI in the LF group. - Clinical controls and BDI: no significant differences in either group. - Resting pain: significant decrease in the LF group. - FM symptoms: improvement in sleep quality, reduction in morning stiffness, improvement in general health questionnaire, improvement in health assessment questionnaire and in rheumatologist’s global questionnaire in the LF group. - Exercise test and handgrip power: no significant changes in any group.
Donaldson M S, 2001 [8]	Observational cohort study	30 persons with FM (28 female and 2 male) (average 45–54 years)	- Pure vegetarian diet mainly raw	- Physical performance: significant improvements. - Hand grip strength: significantly decreased. - Impact of FM (FIQ): significantly reduced. - Quality of life (QOLS): significant improvements in active recreation, health, socialization, and participation in organizations. - Health Survey (SF-36): Significant improvement in physical functioning, physical role, general health, vitality, social functioning, emotional role, and mental health, except in bodily pain. Significant improvement in 7 of these 8 areas, with bodily pain being the exception.
Martínez-Rodríguez A, 2018 [36]	Randomized clinical trial	21 females with FM (34 ± 3 years)	- Intervention group A (*n* = 7); rehabilitation program focused on core stabilization and isocaloric lacto-vegetarian diet. - Intervention group B (*n* = 7); placebo rehabilitation program and isocaloric lacto-vegetarian diet. - Control group C (*n* = 7); no rehabilitation program and isocaloric omnivorous diet.	- Body composition: fat-free mass increased significantly in group A, no differences were found in group B and there was a significant decrease in group C. Fat mass decreased significantly in group A, was unchanged in group B and increased significantly in group C. - VAS scale: decreased significantly in group A, no significant change was found in group B and increased in group C.
Michalsen A, 2005 [39]	Observational cohort study	51 patients with RA (9 female) or FM (32 female and 3 male). The 4 groups: FM and fasting (*n* = 21, 52.0 ± 10.0 years); RA and fasting (*n* = 21, 57.6 ± 6.5 years); FM and vegetarian diet (*n* = 14, 51.6 ± 13.3 years); RA and vegetarian diet (*n* = 7, 49.4 ± 14.3 years)	- Fasting intervention. - Diet intervention: normocaloric mostly vegetarian modified whole grain Mediterranean diet. - Additional treatments: physical exercise, physiotherapy, stress program and different concomitant therapies such as hydrotherapy and massage for all participants.	- Body weight: decreased in fasting patients compared to patients on a vegetarian diet. - Symptom severity: no significant variation among FM patients. - Stool analysis: no significant change in either group.
Hanninen O, 2000 [38]	Clinical trial	115 persons: 40 healthy volunteered, 33 FM patients, 42 RA subjects. They were divided into LF and omnivorous controls	- “LF diet” (uncooked vegan diet) - Control (omnivorous diet)	- Antioxidant and lignan levels: significantly increased in subjects on the LF diet. - Rheumatoid symptoms: Significant subjective and objective relief of symptoms with the LF diet. Improvement on joint stiffness and general health status. - VAS scale: significant differences were found, subjects who adopted the LF diet improved the punctuation in this scale.
Hostmark A T, 1991 [37]	Clinical trial	8 female and 2 males with FM (49.9 ± 4.1 years)	- Vegetarian diet	- Body weight: significantly decreased. - Well-being: no significant differences were found, although 7 participants did improve subjectively. - Laboratory parameters—cholesterol, peroxides, fibrinogen, triacylglycerol: Significant decrease in serum peroxides, plasma fibrinogen concentration, serum total cholesterol and apolipoprotein A and B levels. There was no significant alteration in mean serum triacylglycerol concentration.

BDI: Beck depression inventories; BMI: body mass index; ESR: erythrocyte sedimentation rate; FIQ: fibromyalgia impact questionnaire; FM: fibromyalgia; LF: living food; QOLS: quality of life survey; RA: rheumatoid arthritis; SF-36: short form health survey; VAS: visual analogue scale.

## Data Availability

The data presented in this study are available on request from the corresponding author.

## Supplementary Materials

The following are available online at https://www.mdpi.com/article/10.3390/ijerph18094955/s1, Table S1: assessment of the methodological quality of the observational studies included in the review, Table S2: assessment of the methodological quality of the clinical trials studies included in the review, Table S3: assessment of the methodological quality of all studies included in the review.
