# Supplementary material for: Vegetarian and Vegan Diet in Fibromyalgia: A Systematic Review
Source: Int J Environ Res Public Health. 2021 May 6;18(9):4955. doi: 10.3390/ijerph18094955 (PMC8125538; doi:10.3390/ijerph18094955)
Supplement: Supplementary file 1 [file ijerph-18-04955-s001.zip › ijerph-1196943-supplementary.pdf]

Supplementary Table S1. Assessment of the methodological quality of the observational studies included in the review.

STROBE Checklist (Erik vom Elm 2008) – for cohort studies "yes" "n/a" "no"																						
**ITEM**	**1**	**2**	**3**	**4**	**5**	**6**	**7**	**8**	**9**	**10**	**11**	**12**	**13**	**14**	**15**	**16**	**17**	**18**	**19**	**20**	**21**	**22**
**Donaldson M S, 2001**	yes	yes	no	yes	yes	yes	yes	yes	no	yes	yes	yes	yes	yes	yes	yes	yes	yes	yes	yes	yes	yes
**Michalsen A, 2005**	yes	yes	no	no	yes	yes	yes	yes	no	yes	yes	yes	no	yes	yes	n/a	n/a	no	yes	yes	no	no

n/a: not appear or not applicable

Supplementary Table S2. Assessment of the methodological quality of the clinical trials studies included in the review.

PEDro Scale (Natalie A de Morton 2009) – for clinical trials studies "yes" "n/a" "no"											
**ITEM**	**1**	**2**	**3**	**4**	**5**	**6**	**7**	**8**	**9**	**10**	**11**
**Martínez-Rodríguez A, 2018**	yes	yes	yes	n/a	no	no	no	no	n/a	yes	yes
**Kartinen K, 2000**	yes	n/a	no	n/a	no	no	no	yes	yes	n/a	yes
**Hanninen O, 2000**	no	n/a	no	n/a	no	no	no	yes	yes	n/a	no
**Hostmark A T, 1991**	no	n/a	no	n/a	no	no	no	yes	yes	n/a	no

n/a: not appear or not applicable

Supplementary Table S3. Assessment of the methodological quality of all studies included in the review.

MIXED METHODS APPRAISAL TOOL (MMAT) (Pluye P 2011) – "yes" "n/a" "no"							
**ITEM**	**S1**	**S2**	**1**	**2**	**3**	**4**	**5**
**Donaldson M S, 2001**	yes	yes	no	yes	yes	n/a	yes
**Kaartinen K, 2000**	yes	yes	yes	n/a	yes	n/a	yes
**Hanninen O 2000**	yes	yes	n/a	yes	yes	no	yes
**Michalsen A, 2005**	yes	yes	n/a	yes	yes	yes	yes
**Hostmark A T, 1991**	yes	yes	no	yes	yes	n/a	yes
**Martínez-Rodríguez A, 2018**	yes	yes	Yes	Yes	Yes	No	yes

n/a: not appear or not applicable
